# Association of Brucella Meningoencephalitis with Cerebrospinal Fluid Shunt in A Child: A Case Report

**Published:** 2013

**Authors:** Babak ABDINIA, Mohammad BARZEGAR, Majid MALAKI, Haleh BEHBOD, Shahram OSKOUI

**Affiliations:** 1 Assistant Professor, Pediatric Health Research Center, Departments of Infectious diseases, Tabriz University of Medical Sciences, Tabriz, Iran; 2 Professor of Pediatric Neurology, Departments of Pediatric Neurology, Tabriz University of Medical Sciences, Tabriz, Iran; 3 Assistant Professor, Departments of Nephrology Diseases, Tabriz University of Medical Sciences, Tabriz, Iran; 4 Resident of Pediatric, Tabriz University of Medical Sciences, Tabriz, Iran; 5 Assistant Professor, Departments of Infectious Diseases, Tabriz University of Medical Sciences, Tabriz, Iran

**Keywords:** Brucella, Meningoencephalitis, Shunt

## Abstract

**Objective:**

Brucellosis is an endemic zoonosis in Iran. It is a systemic infection that can involve any organs or systems of the body and have variable presentations.

Ventriculoperitoneal (VP) shunt infections due to brucellosis have been rarely reported in the literatures.

This is the history of a four years old boy who developed Brucella meningoencephalitis at the age of 42 months, whilst he had a VP shunt in situ for hydrocephalus treatment. Also, he presented brucellosis as acute abdomen. This patient was treated with trimethoprim-sulfamethoxazole, gentamicin and rifampicin. The shunt was extracted and all clinical and laboratory test abnormalities subsided through this management.

We propose that in a patient with Brucella meningoencephalitis, the cerebrospinal fluid shunt system can be extracted and treatment with appropriate combination of antibiotics could be successful. Moreover, it shows that brucellosis should be considered in the differential diagnosis for acute abdomen and ascites in endemic regions.

## Introduction

Brucellosis is an enedemic zoonosis in Iran that is mostly caused by Brucella melitensis subgroup (1).

Ventriculoperitoneal shunt infection because of brucellosis is a rare phenomenon (2). Despite the high incidence of brucellosis in Iran, that is probably due to the widespread use of Fresh cheese (22.4%), animal husbandry (11.3%), laboratory worker (8.1%) and veterinary profession (1.5%), neurological complications of brucellosis occur in up to 0.6% of affected patients (1). The various presentations of brucellosis due to different organs involvement by this bacterium need to establish new management methods for uncommon presentations of brucellosis such as neurologic complication.

## Case Report

A 6-month-old boy with hydrocephalus was referred to our hospital in 2008. The hydrocephalus during the first admission was confirmed by computed tomography (CT) scan. Examination of cerebrospinal fluid (CSF) did not reveal any abnormality.

A VP shunt was inserted in June 2008 and the infant was discharged in acceptable condition, with good control of hydrocephalus confirmed by another CT scan on discharge day. Approximately 16 months after insertion of the VP shunt, the child was readmitted with a one-week history of fever (up to 38.5°C), and acute abdominal pain. The diagnosis was brucellosis based on serum agglutination test 1/640 and 1/1280 three days later. A titre of 1/320 measured in 2 mercaptoethanol (2ME) test. The patient was treated with trimethoprimsulfamethoxazole, rifampin, and gentamicin. His blood culture was negative.

The Ascitic fluid examination showed a glucose level of 89 mg/dL, a protein level of 5800 mg/dL, white blood cell (WBC) count was 480 (10% polymorphonuclear cells and 90% mononuclear), and the red blood cell (RBC) count was 4800.

After 4 months, he was again admitted to our hospital with drowsiness, vomiting and fever. Clinical diagnosis was suggestive for a VP shunt infection. Laboratory results were as follows: Hemoglobin, 9.1 g/dL; WBC count, 6.1×103 cells/mm3. 

A sample of ventricular CSF analyzed was slightly xanthochromic and contained a total of 4000 cells (70% polymorphonuclear cells and 30% lymphocytes), 80 mg/ dL glucose, and 192 mg/dL protein. No organisms were observed on the direct gram-stained smear. 

Blood culture was sterile and CSF serological test was positive (Brucella agglutinin titer was 1/80).

**Figure 1:**
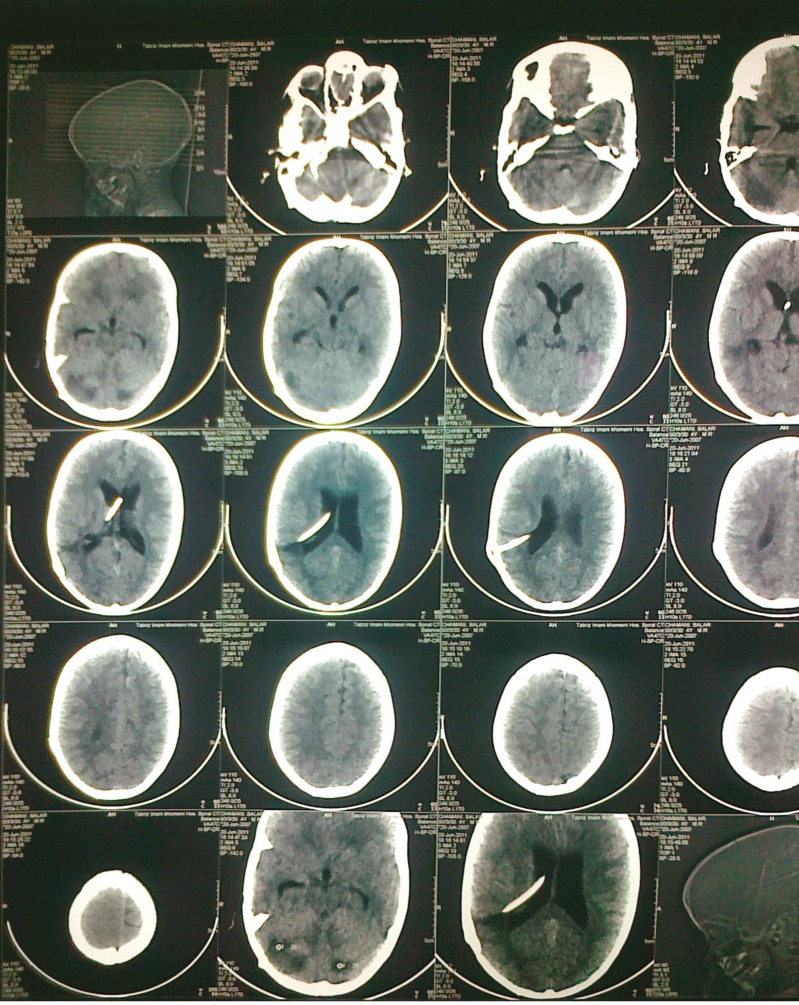
Brain CT scan before extraction of ventriculoperitoneal shunt

**Figure 2:**
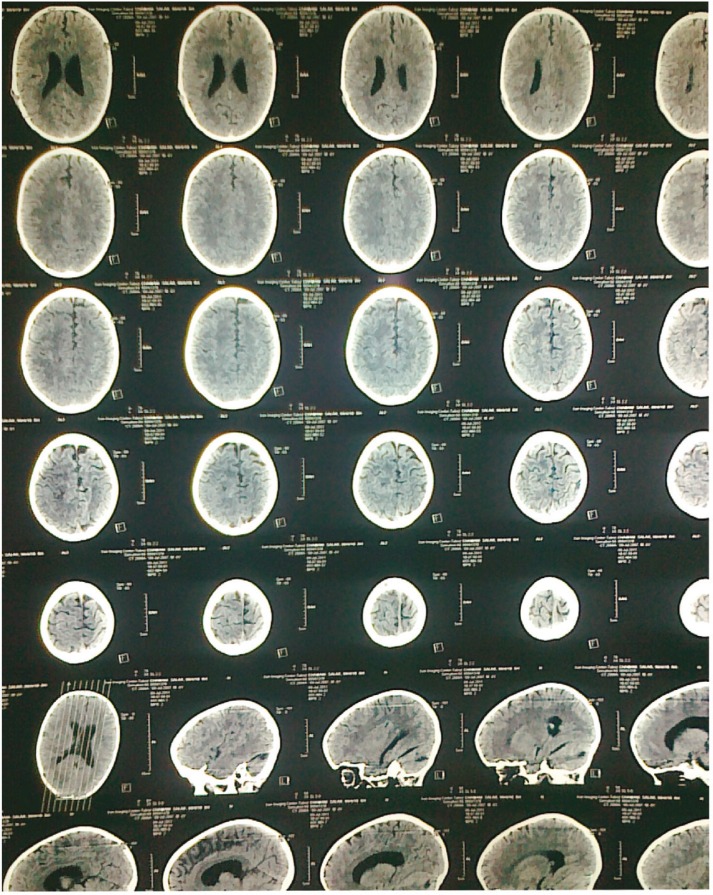
Brain CT scan after extraction of ventriculoperitoneal shunt, there was no changes in hydrocephalus

The patient responded well to a course of rifampin, gentamicin and trimethoprim-sulfamethoxazole daily. Three days later the shunt was extracted. He was well on three follow-up visits at the outpatient clinic and the latest follow-up visit was 12 months after the diagnosis has been made. Figure 1 and 2 show Brain CT scan before and after extracting the VP shunt, respectively.

## Discussion

Brucellosis should be always kept in mind in the differential diagnosis of patients with prolonged fever (3).The most frequent isolated species among the Brucella group of pathogens in Iran is Brucella melitensis. The high incidence of brucellosis in Iran is possibly because of the widespread use of Fresh cheese (22.4%), laboratory worker (8.1%), animal husbandry (11.3%), and veterinary profession (1.5%) (1). Childhood brucellosis is uncommon (4). it is usually mild and self-limiting. neurobrucellosis presenting as meningitis or meningoencephalitis is rarely occurred (5) The association of Brucella meningitis with a previously inserted VP shunt in a young child is a rare occurrence. The patient responded well to a combination of trimethoprim-sulfamethoxazole, rifampin and gentamicin, a regimen that has been reported on because of the additional bactericidal ability of rifampicin to penetrate the cells’ interiors in the reticuloendothelial system, wherein the Brucella organisms reside (6)

The presence of a VP shunt system in our patient with Brucella meningoencephalitis, confronted us with a difficult decision as to whether remove the VP shunt. In patients with bacterial ventriculitis, it is wise to remove the VP shunt system (4). However, on the contrary, there are views that recommend intrathecal and systemic antibiotics for decontamination of the shunt tubing systems and control the CNS and/or systemic infection (8).

We suggest extraction of the VP shunt while the patient received combination Antibiotic therapy. This resulted in amelioration of the clinical symptoms and signs as well as normalization of the CSF abnormalities. The child remained well in follow-up 12-months later.

**In conclusion, **in patients with Brucella meningoencephalitis, we recommend that the CSF shunt system can be extracted while combination drug therapy is given.
